# Rethinking Childhood-Onset Hypertrophic Cardiomyopathy: A Review of Molecular Mechanisms and Unique Therapy Considerations

**DOI:** 10.3390/jcdd12100374

**Published:** 2025-09-23

**Authors:** Caitlin Menzies, Vernon W. Dolinsky

**Affiliations:** 1 Diabetes Research Envisioned and Accomplished in Manitoba (DREAM) Theme of the Children’s Hospital Research Institute of Manitoba, Winnipeg, MB R3E 3P4, Canada; 2 Department of Pharmacology and Therapeutics, Rady Faculty of Health Science, College of Medicine, University of Manitoba, Winnipeg, MB R3E 3P4, Canada

**Keywords:** hypertrophic cardiomyopathy, molecular genetics, children, personalized medicine, cardiology

## Abstract

Childhood-onset hypertrophic cardiomyopathy (HCM) is a cardiac disorder presenting unique diagnostic and therapeutic challenges in children that require tailored clinical attention compared to HCM arising at other life stages. Despite this, current treatment strategies specific to childhood-onset HCM are lacking and are predominantly extrapolated from adult-specific treatment guidelines. This review explores the molecular basis, clinical implications, and management strategies specific to childhood-onset HCM. Advances in molecular genetics have elucidated diverse pathogenic pathways and genotype-phenotype correlations, revealing age-specific disease modifiers distinct from adult-onset forms. Current management includes pharmacologic, surgical, and device-based interventions, tailored to individualized needs. However, there is a lack of evidence for the efficacy and safety profiles of these treatments in children, meaning children may be receiving sub-optimal care. Emerging approaches, such as gene-targeted therapies and precision medicine frameworks, show promise, but require further investigation. Enhancing early diagnosis and personalized care is crucial for improving outcomes and reducing long-term disease burden in affected children. This review underscores the necessity for specific research to refine risk stratification and treatment paradigms for childhood-onset HCM.

## 1. Introduction

Hypertrophic cardiomyopathy (HCM) is a disease of the cardiac muscle characterized by abnormal thickening of the myocardium [1,2,3,4,5,6,7,8]. Although some individuals with HCM remain asymptomatic, structural abnormalities may impair the heart’s ability to pump blood efficiently and increase the risk of arrhythmias, heart failure, and sudden cardiac death (SCD). While HCM is often associated with adults, its onset also occurs during childhood. Childhood HCM presents unique challenges in diagnosis, risk stratification, and treatment due to the variability in disease progression and limited childhood-specific clinical guidelines and research [2,3,4,9,10]. Studying HCM in childhood is critical, as early diagnosis and tailored management can significantly influence disease progression and long-term outcomes. This paper explores the molecular basis of childhood-onset HCM, highlighting genetic and cellular mechanisms involved in disease development, and also reviews current and emerging strategies for its clinical management.

## 2. HCM Across the Lifespan

While HCM can develop at any point across the lifespan, it is most commonly diagnosed during three key periods: infancy, childhood, and adulthood [1,8]. Childhood-onset HCM is defined as unexplained left ventricular hypertrophy diagnosed between 1 and 18 years of age [1]. Childhood-onset HCM is not simply an early presentation of adult disease, but rather a clinically and biologically distinct entity with unique underlying etiology, disease trajectory, and treatment response [8,11]. As a result, management strategies must be tailored not only to disease severity but also to the unique physiological and genetic landscape of children. To optimize care, HCM patients are categorized into distinct groups based on key clinical and biological factors, with age of onset being one of the most informative classification strategies [1,5,8].

### 2.1. Adult-Onset HCM

**Figure 1:**
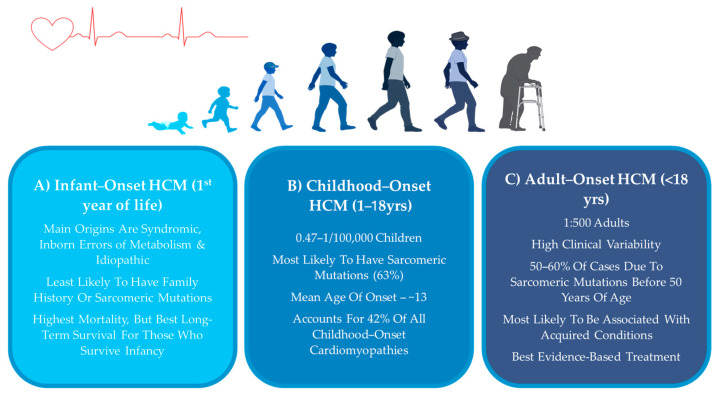
Summary of key clinical characteristics of hypertrophic cardiomyopathy (HCM) in (**A**) infants (1st year of life) (**B**) children (1–18 yrs) and (**C**) and adults (<18 yrs). Figure created with BioRender.com (accessed on 15 June 2025).

Adult-onset HCM (Figure 1C) refers to cases which manifest in adulthood, specifically after the age of 18. Unlike childhood-onset or infantile-onset HCM, which often presents severe symptoms early in life, adult-onset HCM may remain asymptomatic for years before leading to detectable clinical complications [1,2,12]. The estimated prevalence of HCM is 1:500 in the adult population [1,2,4,9,12,13,14,15]. This disorder is more likely to be associated with acquired cardiovascular disorders, such as high blood pressure, diabetes, and obesity, but are also commonly genetically derived [4,7,15,16]. Compared to childhood-onset, adult-onset HCM often progresses more gradually [4,16]. However, adult patients face elevated risk of arrhythmia, stroke, and heart failure, making early diagnosis and tailored management essential [9].

### 2.2. Infantile HCM

Infantile-onset HCM (Figure 1A) is diagnosed within the first year of life and is relatively rare, but clinical presentation is often extreme, with symptoms such as heart failure, respiratory distress, hypotonia, or feeding difficulties [1,16]. Reports vary regarding the contribution of sarcomeric gene mutations; while some studies suggest that infantile HCM is most commonly associated with inborn errors of metabolism, such as Pompe disease, mitochondrial disorders, or certain syndromic conditions [1,2,7,12,14], other works report a substantial burden from sarcomeric mutations in this age group [17], highlighting the need for larger studies to clarify the predominant etiologies of infantile HCM. Moreover, idiopathic cases may conceal unidentified genetic etiologies. The prognosis in infantile HCM is generally poor, particularly when associated with metabolic or mitochondrial disease. Infantile HCM is associated with high initial mortality and morbidity rates; however, survival rates dramatically improve for those who survive beyond the first year of life and are often more favorable compared to those diagnosed in childhood [1,2,16,18]. Infants diagnosed with HCM due to inborn errors of metabolism or malformation syndromes face significantly higher mortality risks than other etiologies, with five-year survival rates of 41.7% and 74.4%, respectively, compared to 82.2% for infantile idiopathic HCM and 93.9% for noninfantile idiopathic HCM [2]. While SCD is the most common cause of mortality in children, infant deaths are more commonly attributable to heart failure [7,8]. Etiology is important when guiding treatment. Since a major underlying factor in this population is the high degree of inborn errors of metabolism, specialized treatments such as enzyme replacement therapies have shown efficacy in this population [2,7,8]. Given the frequent autosomal recessive or mitochondrial inheritance patterns, genetic counseling is essential for family planning and risk assessment in siblings [2,15].

### 2.3. Childhood-Onset HCM

Childhood-onset HCM (Figure 1B) is typically identified in adolescence at an estimated prevalence of 0.47–1 per 100,000, accounting for 42% of all childhood-onset cardiomyopathies [1,2,7,9,12,13,16,18,19,20]. Compared to adults, a broader range of causes have been reported to be involved in children, including sarcomeric gene mutations, metabolic disorders, and syndromic conditions, which influence prognosis and therapeutic decisions. Additionally, developmental differences in children—such as age-dependent variations in metabolic rate, drug metabolism, and organ maturity—impact both disease expression and response to therapy [1,2,5,7,8,18]. Mortality in older children remains rare; however, those diagnosed in childhood are twice as likely to require heart transplantation or a ventricular assist device compared to adult-onset cases, underscoring the increased severity of early-onset disease [1,2].

## 3. HCM Symptoms in Children

Across all ages, HCM presents with symptoms related to dyspnea, chest pain, palpitations, syncope, fatigue, or, in severe cases, SCD [2,19]. There is a wide spectrum of symptom severity in childhood, ranging from asymptomatic cases detected through family screening to profound symptoms [2,4,15,18,19]. Symptom presentation can be challenging to interpret, as fatigue, dizziness, or chest discomfort may be vague and mistaken for benign conditions such as asthma or anxiety [2,11,18]. Compared to adults, children with HCM are at greater risk of life-threatening ventricular arrhythmias and SCD, which are a significant cause of adverse events [8,9,13]. However, childhood HCM is typically associated with lower incidence of heart failure and atrial fibrillation in early disease stages compared to the early disease stages of adults [1,13]. As individuals age, a diagnosis of HCM in childhood is associated with nearly half experiencing a major cardiac event 25 years following diagnosis [1,7]. Childhood-onset HCM is also often associated with left ventricular outflow tract (LVOT) obstruction, where severe hypertrophy blocks blood flow from the left ventricle to the aorta. In adults, LVOT obstruction severity is correlated with symptom severity; however this association appears weaker in children, though it still contributes to long-term risk [4]. Nonetheless, LVOT obstruction can contribute to diastolic dysfunction, arrhythmia, and increased risk of SCD [2,4,19]. HCM-related SCD occurs more frequently in children than adults and is more likely to present as their first clinical event, often during physical activity [4,18]. As HCM is a progressive condition, symptoms can worsen over time, requiring lifelong monitoring [2,7,19]. In fact, progression of left ventricular hypertrophy may be more rapid in children than in adult populations [4,7]. Given these risks, children with HCM should receive close clinical monitoring regardless of symptom presence, especially during periods of rapid growth or increased physical exertion.

## 4. Molecular Pathophysiology of Childhood-Onset HCM

### 4.1. Physiological vs. Pathological Cardiac Hypertrophy

Cardiac hypertrophy can occur in response to various stimuli and can be physiological (e.g., exercise) or pathological (e.g., neurohormonal excess) in nature [5,21,22,23,24]. Physiological hypertrophy is a beneficial and adaptive form, seen in athletes or pregnancy, where the heart enlarges proportionally to the initiating stimulus to maintain cardiac output in response to an increase in workload. Importantly, physiological hypertrophy is a reversible form of cardiac growth that is supported by adaptive mechanisms—like enhanced angiogenesis—that ensure sufficient oxygen and nutrient delivery to the heart [21]. Pathological hypertrophy arises in response to chronic stress and initially maintains cardiac output. However, in the absence of adaptive mechanisms—such as adequate angiogenesis to support nutrient and oxygen delivery—this response becomes maladaptive over time [5,21,22,23,24]. The sustained stress leads to structural and molecular changes, including fibrosis and apoptosis, ultimately impairing cardiac function irreversibly [4,21,22,25]. Children, especially adolescents, may develop physiological hypertrophy from high levels of physical activity, which overlaps in appearance with early pathological HCM. Standard diagnostic tools are not always reliable in differentiating physiological from pathological hypertrophy in children. Differentiation is especially important in children because misdiagnosis can have serious psychosocial and clinical consequences.

In both physiological and pathological forms of hypertrophy, the increased workload on the heart translates into mechanical stress sensed at the molecular level, where sarcomere stretch and neurohormonal activation triggers intracellular signaling cascades that promote hypertrophic growth [21,22,23]. Because cardiomyocytes are terminally differentiated and non-proliferative, the heart compensates by enlarging existing cells through increased protein synthesis, cell growth, and structural remodeling to enhance the physical size, and thus the contractile force of cardiomyocytes to improve cardiac output with each heartbeat [5,20,21,22,23,25]. Physiological hypertrophy is primarily regulated by the PI3K-AKT-mTOR pathway, supporting adaptive remodeling, while pathological hypertrophy is driven by maladaptive signaling through pathways such as calcineurin-NFAT, MAPK, and adrenergic signaling [5,6,20,21,22].

Interestingly, many current treatments for HCM do not directly target these molecular pathways but instead focus on symptom alleviation. Symptom-directed therapies are justified through immediate relief and clinical benefits related to morbidity and mortality. Investigation into signaling-related therapies is still in early stages, and the evaluation of their efficacy and safety compared to standard treatments remains uncertain. Moreover, because HCM arises from a wide spectrum of origins, and because a significant portion of patients have no identifiable genetic cause, focusing solely on molecular interventions risks excluding many patients. Moreover, the disease involves many interconnected pathways that are not yet fully understood, and even less is known about how these pathways may differ between children and adults, making it difficult to predict how a single molecular target will influence disease progression across age groups. In sum, research into signaling cascades to understand the pathophysiology and potential therapeutic targets of HCM is a warranted area of investigation, but a greater body of research is required to test the benefit of these approaches against current benchmark treatment strategies [6,18].

### 4.2. Sarcomeric and Non-Sarcomeric HCM

Childhood-onset HCM are a heterogeneous group of disorders with a range of underlying causes. These include pathogenic variants in genes encoding sarcomeric proteins, malformation syndromes (e.g., Noonan syndrome), inborn errors of metabolism (e.g., Pompe disease), and neuromuscular disorders (e.g., Friedreich ataxia) [1,2,8,12].

#### 4.2.1. Sarcomeric HCM

The sarcomere is the basic functional unit of the heart that depends on the coordinated action of sarcomere proteins (actin, myosin, troponin, and tropomyosin) to facilitate a synchronized, wave-like contraction of millions of cardiomyocytes which effectively enables pumping of blood into the circulation as well as the subsequent relaxation and refilling of the heart [4,5,20,21,22,25,26]. Given the central role of the sarcomere in coordinating effective contraction and relaxation cycles, mutations in sarcomere genes can disrupt cardiac function and ultimately drive hypertrophic remodeling. Genetic screening revealed pathogenic or likely pathogenic sarcomeric variants in 64% of childhood-onset cases, compared to 49% in adults and 36% in infants [1,2,5,25,26].

**Figure 2:**
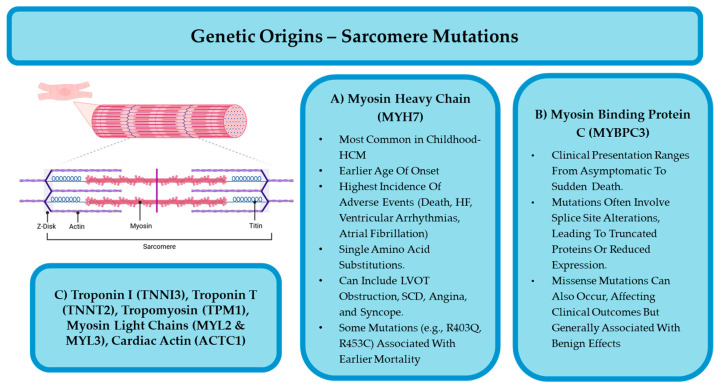
Summary of the most implicated sarcomeric mutations—including (**A**) MYH7 and (**B**) MYBPC3—and their associated clinical features. Less common sarcomeric mutations (**C**) (TNNI3, TNNT2, TPM1, MYL2, MYL3, ACTC1) are also listed. Figure created with BioRender.com (accessed on 15 June 2025).

Sarcomeric HCM is diagnosed in the presence of a pathogenic or likely pathogenic sarcomeric gene mutation and is typically inherited in an autosomal dominant pattern, though some cases arise without a clear family history due to de novo mutations or incomplete penetrance [2,4,5,9,15,16,18,24,26]. The most frequently affected genes are MYH7 (beta-myosin heavy chain) (Figure 2A) and MYBPC3 (myosin-binding protein C3) (Figure 2B), which together account for approximately 70% of variant-positive cases, while other sarcomeric gene mutations (TNNI3, TNNT2, TPM1, MYL2, MYL3, ACTC1) (Figure 2C) each contribute to a smaller proportion (1–5%) of cases [1,4,5,6,7,15,16,19,20,24,25,26,27].

MYH7 mutations, which primarily involve single amino acid substitutions affecting force-generating regions, are associated with an earlier onset of disease and a higher incidence of adverse cardiac events and SCD [5,14,17,26,27]. MYBPC3 mutations, in contrast, often involve splice-site alterations leading to truncated proteins or reduced expression, with missense mutations generally having more benign effects [5,20,24,26,27]. MYBPC3 are the most common variant in adult-onset cases [1,5]. Some studies have found that mutations in thick filaments (particularly in MYH7 and MYBPC3) are especially associated with poor outcomes in children with HCM [9]. Other sarcomeric mutations, such as those in troponin proteins, are linked to varied phenotypes—TNNI3 (troponin I) mutations are associated with altered cardiac shape but low SCD risk, while TNNT2 (troponin T) mutations carry a high SCD risk despite mild hypertrophy [5,14,24,27]. Troponin C and tropomyosin mutations have been linked to early-onset HCM with severe hypertrophy and high childhood SCD incidence, respectively [5,27].

When considering genetic subtypes within childhood onset HCM, sarcomeric HCM is associated with severe outcomes relative to non-sarcomeric forms [1,17]. Sarcomeric variants contribute to a 63% higher risk of major cardiac events in childhood-onset HCM, particularly driven by heart failure and atrial fibrillation [1]. Thus, although children with HCM generally have lower early-stage risk of heart failure and atrial fibrillation than adults, sarcomeric mutations still confer higher risk within the childhood population [1,13]. Interestingly, the impact of sarcomeric mutations differs by age of onset. While childhood-onset sarcomeric HCM is strongly associated with elevated heart failure risk, adult-onset sarcomeric HCM is more clearly linked to all-cause mortality and atrial fibrillation [1]. Despite advances in genetic characterization, the mechanisms explaining how these mutations contribute to the HCM phenotype remain incompletely understood [4,5,24]. Less is known about patients with compound mutations, who may experience severe outcomes, including a higher risk of arrhythmic events or end-stage heart failure, emphasizing the importance of gene-gene interactions in modifying HCM disease progression [2,5,9].

#### 4.2.2. Non-Sarcomeric HCM

Non-sarcomeric causes of HCM are most commonly diagnosed in the youngest pediatric patients and often present with extracardiac manifestations that can aid in identifying the underlying etiology [2,16]. Establishing the cause is critical, as some disease-specific treatments, particularly for inborn errors of metabolism, may significantly alter disease progression, treatment, and prognosis [2,3,14,16]. Idiopathic HCM, the second-most common cause of HCM in children, is diagnosed when no genetic or secondary cause is identified after thorough evaluation [2,3]. As genetic understanding continues to evolve, patients without a known cause are advised to undergo periodic retesting as new pathogenic gene mutations are added to HCM panels and previously classified variants are reassessed [2,5,14,20].

Inborn errors of metabolism that cause HCM include glycogen storage disorders, fatty acid metabolism disorders, and mitochondrial defects. Glycogen storage disorders, such as Pompe disease, Danon disease, and PRKAG2 cardiomyopathy, are characterized by glycogen-filled vacuoles accumulating in multiple tissues, including cardiomyocytes, leading to increased cardiac mass, LVOT obstruction, and impaired cardiac function [3,12,14,20,24]. Lysosomal storage diseases, including mucopolysaccharidoses, result from the accumulation of undigested or partially digested macromolecules, causing cellular dysfunction and organomegaly, with some forms leading to HCM [12,14,20]. Mitochondrial disorders stem from mitochondrial respiratory chain dysfunction, affecting organs with high energy demands, particularly the heart. Several mitochondrial syndromes and fatty acid oxidation disorders involve myocardial dysfunction, with HCM being the most common form of mitochondrial-associated cardiomyopathy [2,12,14]. Cardiac contractility and relaxation are intrinsically related to calcium homeostasis; mutations in calcium channel proteins as well as proteins known to influence calcium homeostasis, including phospholamban junctophilin 2, and the CACNA1C-encoded CaV1.2 calcium channel, have been implicated in HCM, albeit at a lesser degree in children than in adults [5,6,15,22,24,25,28]. Finally, in children HCM is often associated with syndromes, such as RASopathies, which can complicate management due to multisystem involvement. These syndromes may present with additional clinical features beyond the heart, often require more individualized care compared with those who have isolated, non-syndromic HCM [12].

## 5. Diagnostic Considerations in Children

Given the considerable range in which children may display symptoms, HCM is typically diagnosed in children when symptoms arise or through screening after a first-degree relative is diagnosed or experiences sudden unexplained death [14,17,18,29]. However, many cases are detected incidentally during routine medical visits when a heart murmur or abnormal rhythm prompts an electrocardiogram (ECG) examination, where electrical activity of the heart is measured to understand the timing and strength of cardiac contractions [4,14,20,29]. While an ECG can reveal electrical abnormalities in cases of severe hypertrophy, it may not be sensitive enough to detect early or mild forms of the disease [4,5,6,28].

The primary diagnostic tool for HCM is echocardiography (ECHO), which provides real-time imaging of the heart using high-frequency sound waves [2,7,13,14,15,16,25]. In adults, HCM is diagnosed based on a maximal end-diastolic wall thickness of ≥15 mm, with 13–14 mm considered diagnostic in individuals with a positive family history or genetic mutation [2,4,5,13,18,19]. In children, however, wall thickness must be adjusted for body size using Z-scores, which compare the child’s heart measurements to population norms [2,5,7,16,19]. A diagnosis is made when wall thickness exceeds 2 standard deviations from the predicted mean in children with a family history or genetic mutation and 2.5 standard deviations in those without [2,5,7,19]. However, there are limitations in this method as specific Z-score thresholds have not been independently standardized in pediatric populations, leading to some diagnostic uncertainty. Additionally, intense physical activity may cause some physiological hypertrophy, potentially mimicking pathological HCM. While echocardiography cannot reliably differentiate between the two, hypertrophy due to exercise rarely exceeds a Z-score of 2.5–3. To improve diagnostic accuracy, a temporary restriction from intense physical activity for six months before evaluation may be recommended [2,4,5,16,17,18]. Subtle abnormalities in diastolic function may also hint at pathological hypertrophy as opposed to physiological hypertrophy, this however is not required for diagnosis [2,4].

It is recommended that children who meet diagnostic criteria for HCM undergo routine longitudinal monitoring to detect subtle new clinical symptoms, including periodic ECG, ECHO, and exercise testing every 1–2 years in preadolescents and annually during adolescence [18,19]. Periodic cardiac magnetic resonance imaging (MRI) evaluation of late gadolinium enhancement (LGE), as evidence of myocardial fibrosis is recommended, although the frequency of testing and the threshold level of fibrosis that represents a risk factor for sudden death remain uncertain in children given the limited outcome data [15,19,30].

## 6. HCM Treatment in Children

Although there are evidence-based, goal-directed treatment algorithms that are often updated by various associations for the treatment of adult-onset HCM, there is a significant lack of research into specific HCM treatment for children [7,8,14,19]. Current guidelines for childhood-onset HCM are largely based on expert consensus and extrapolated from adult data, as there are few studies specifically looking at the effectiveness of treatment and effects on long-term outcomes in children [7,8,10,14,19]. Using adult data may overlook age-specific nuances and could result in inaccurate diagnoses, inappropriate treatments, or overlooked complications that are unique to the children [19]. Here, we will review current treatment strategies in the management of childhood-onset HCM, limitations to these treatments, and gaps in the literature that require future investigation.

### 6.1. First-Line Pharmacological Treatments of Childhood-Onset HCM

The treatment for HCM in children almost always begins with pharmacologic management, most commonly beta-adrenergic antagonists/blockers or calcium channel antagonists/blockers [18,25,31,32]. These agents, by reducing heart rate and contractility, improve coronary flow/demand ratio, decrease LVOT obstruction gradient, improve diastolic filling, and reduce symptoms [25,31].

#### 6.1.1. Beta-Adrenergic Antagonists/Blockers

Beta-adrenergic blockers, also known as beta-blockers, are considered the first-line treatment for HCM in both children and adult populations [3,4,12,13,14,16,18,25,27,29,31,32]. The primary mechanism of beta-blockers is reducing the effects of sympathetic nervous system activation in the myocardium [13]. The therapeutic actions include a reduction in heart rate, prolonged diastole, improved ventricular filling, decreased contractile force, and reduced myocardial oxygen demand, collectively mitigating the hypercontractility characteristic of HCM and limiting further cellular damage [2,3,13,16,19,27]. Moreover, beta-blockers alleviate chest pain, syncope, dyspnea, LVOT obstruction, ventricular arrhythmias, ischemia, and SCD related to HCM [2,13,16,19,29,31].

In contrast to adults, the treatment of children with beta-blockers has no standardized recommended dose. Instead, titration of the dose based on the physiological effect in the individual patient is required in children, as treatment with a high dose relative to adults is often required to achieve therapeutic benefit [2,4,13,16,29]. This is attributed to the age-dependent pharmacokinetics of beta-blockers [13,16]. Even at high doses side effects of beta-blockers are generally uncommon in children. However, the effects of high-dose beta-blockers on physical growth have been mixed, with some studies finding impairments in physical growth whereas others see no effect [2,4,16]. Further consideration is required for children with Noonan/Leopard/Costello spectrum disorders as they are associated with mutations in various metabolizing kinases relevant to beta-blockers, and therefore their metabolism and excretion of drugs may be different [16]. Selective beta-1 adrenergic blockers are preferred over non-selective beta-blockers, as the latter may also inhibit alpha-adrenergic receptors, which play a significant role in vasodilation and blood pressure regulation. Non-selective beta-blockers, such as carvedilol, are contraindicated in children due to the risk of inducing significant hypotension, thereby preventing sufficient beta-blockade in children [16,19,29].

#### 6.1.2. Calcium Channel Blockers

For children who do not respond to beta-blockers or experience negative side effects, calcium channel blockers (CCBs) are considered an alternative first-line therapy for HCM [3,5,13,16,19,25,27,29,31]. CCBs inhibit L-type calcium channels and the movement of calcium into cardiomyocytes. Under normal physiological circumstances, calcium enters the cell and acts on the sarcoplasmic reticulum to initiate release of a large calcium store which can then act on the sarcomere to induce contraction and be taken up again by the sarcoplasmic reticulum to induce relaxation [3]. Much like beta-blockers, CCBs effectively reduce contractile force, lower heart rate, enhance diastolic relaxation and filling of the heart, and ultimately reduce cardiac oxygen demand [3,16,19,27]. They also improve microvascular function and increase myocardial perfusion [19]. Non-dihydropyridine CCBs, such as verapamil, are preferred over dihydropyridine CCBs, because they act on the myocardium and the atrioventricular node, rather than vascular smooth muscle. Non-selective CCBs should not be used in children with risk of hypotension and dyspnea due to potential vasodilatory effects [3,5,16,19,25,27,32]. In general, CCBs are well tolerated in children and side effects are rarely encountered in young patients. However, concerns have been raised about the safety of CCBs in children with severe symptoms and heart failure [2,3,16,19] due to potential increase in risk of heart-failure related death [3,16]. Moreover, CCBs should be avoided in infants younger than 6 weeks due to the risks of bradycardia and hypotension, but are generally well tolerated in young children [13,16].

### 6.2. Second-Line Pharmacological Treatments of Childhood-Onset HCM

When the above first-line therapies fail to achieve the desired therapeutic response, additional medications may be introduced to enhance clinical improvement [31].

#### 6.2.1. Renin–Angiotensin–Aldosterone System (RAAS) Suppressing Agents

In cardiac hypertrophy, the RAAS is activated in response to increased cardiac stress [33,34]. RAAS activation promotes vasoconstriction, sodium retention, and myocardial remodeling, all of which can contribute to the progression of hypertrophy [33,34,35]. Therefore, medications that block the RAAS, such as Angiotensin Converting Enzyme (ACE) inhibitors and Angiotensin Receptor Blockers (ARBs) may be used to limit pathological remodeling and slow the progression of cardiac hypertrophy [16,18,19,20,21,22,23,24,25,26,27,29,30,31,32,33,34,35]. ACE inhibitors, which suppress the RAAS by inhibiting the production of Angiotensin II, improve survival and quality of life in adults with left ventricular hypertrophy, and similar benefits may exist for children—but large, randomized studies in children are lacking [19,33,34,36]. ACE inhibitors have been shown to help prevent harmful ventricular remodeling and improve heart function in children, benefits that may be even greater when combined with beta-blockers, although it is unclear whether the improvements are from the ACE inhibitors themselves or from better blood pressure control [33,36].

In children, the ACE inhibitor enalapril has been utilized for hypertension, congestive heart failure and chronic kidney disease, however its effects on HCM have not been reported [35]. However, its ability to limit hypertrophy in adults suggests it may be effective in children [32,36]. Moreover the pharmacokinetics and pharmacodynamics of enelapril has not been thoroughly investigated in children and is only authorized for use in children over 20 kg of body weight [35]. Children often require higher doses than adults for effective treatment, and how well the drugs work can vary depending on the child’s age, origin of dysfunction, or presence of genetic conditions, however this data is limited, and conclusive clinical recommendations have not been made [33,36]. The safety profile of ACE inhibitors in children appears to be similar to that in adults [33,34,36]. However, there is concern that ACE inhibitors could interfere with cardiac development during a time of rapid heart growth [33]. Because of this, doctors are advised to use them cautiously in young patients, even though early evidence of benefit exists [33,34]. In children, the most commonly reported side effects of ACE inhibitors include hypotension, impaired renal function, and hyperkalaemia. A systematic review of 11 studies involving 1050 pediatric heart failure patients treated with enalapril found that hypotension occurred in 0–19% of cases, renal failure in 0–29%, and hyperkalaemia in 0–13% [36].

ARBs like valsartan have shown improvement in left ventricular mass, diastolic function, and serum biomarkers in pediatric patients without advanced myocardial remodeling [13,37], however ARBs were ineffective in reversing established myocardial hypertrophy or fibrosis [13]. The Valsartan for Attenuating Disease Evolution in Early Sarcomeric Hypertrophic Cardiomyopathy (VANISH) trial showed that administering valsartan early in the course of sarcomeric HCM slowed disease progression compared to placebo, improving measures of cardiac structure, function and remodeling [17,37,38]. Additionally, valsartan was safe and well-tolerated with no instances of treatment withdrawal for drug intolerance, including hypotension, hyperkalemia or renal insufficiency [38]. The VANISH study reported greater benefit for individuals with younger age of treatment initiation, mild phenotypes, and sarcomeric HCM [37,38].

#### 6.2.2. Antiarrhythmic Agents

Antiarrhythmic agents with negative inotropic properties, such as disopyramide, are considered a second-line therapy for HCM and can be used in combination with beta-blockers or CCBs if their administration alone does not produce sufficient therapeutic benefit [2,3,4,5,16,19,25,29,31]. Disopyramide primarily functions by blocking sodium channels in cardiac cells, reducing sodium influx during depolarization [2]. This decreases calcium entry into the cells, which subsequently lowers the force of myocardial contraction [2,19]. Additionally, disopyramide is believed to stabilize cardiomyocyte membranes, promoting cellular health and the propagation of electrical signals. While the of disopyramide has been limited in children, it has shown efficacy in managing LVOT obstruction in HCM patients [2]. However, evidence regarding its impact on mortality and ventricular arrhythmias in HCM remains conflicting [2]. Disopyramide has a relatively favorable side-effect profile, with common vagolytic side effects such as dry mouth, blurry vision, and constipation, typically managed with cholinesterase inhibitors [2,16,29]. Large multicenter registries have confirmed their safety and efficacy, supporting its routine use in outpatient settings [19]. A notable potential side effect is the prolongation of the QT interval, which can lead to dangerous ventricular arrhythmias [3,19]. Furthermore, disopyramide has been shown to accelerate atrioventricular nodal conduction, limiting its use as a first-line therapy [3,19,29].

### 6.3. Surgical Interventions for Pediatric HCM

When pharmacological intervention fails to halt the progression of cardiac hypertrophy, surgical interventions are considered to alleviate symptoms and lessen the likelihood of SCD [31]. Surgery is considered the primary treatment for obstructive HCM in children who do not respond to medication [13,31]. Examples include implantable cardioverter-defibrillators (ICDs), septal myectomy and septal ablation. ICD placement remains the most effective strategy for preventing SCD in children identified as high-risk, as pharmacological treatment has shown mixed results specifically in reducing incidence of SCD [4,8,13,18,31,39]. ICDs have also shown efficacy in reducing arrhythmias and mortality rate in children with HCM from 6% to 0.5% per year [13,18,39].

When patients have a large LVOT obstruction gradient and severe symptoms that are unresponsive to pharmacological therapy, septal myectomy to surgically remove a portion of the thickened interventricular septum is performed to relieve LVOT obstruction [4,19,29,31]. Multiple studies have shown over 95% survival rate of pediatric populations years following the procedure. Children are more likely than adult patients to have LVOT obstruction reoccurrence following surgical intervention, however the reason for this phenomenon is not fully understood [2,4,13,16,19]. Moreover, children are at an increased risk of surgical complications due to the size of their aortic valve, making the operation more difficult to perform in small children [4,13]. As an alternative to septal myectomy, septal ablation is a minimally invasive procedure that uses high temperatures or alcohol to intentionally damage and shrink part of the thickened septal wall, reducing LVOT obstruction [2,13,14,16,19,32]. This method is most effective for patients with mild septal hypertrophy, though its use in children is limited due to the high risk of complications, including atrioventricular block and a significant rate of ICD implantation [13].

### 6.4. Lifestyle Management and Exercise Recommendations in Children with HCM

Exercise may exacerbate HCM symptoms and trigger exercise-induced SCD in children who engage in high-intensity sports [2,5,8,14]. Historically, strict sports restrictions were enforced to prevent exercise-induced SCD, but with a growing understanding of the importance of exercise for cardiovascular health and psychosocial well-being, there has been a shift in focus to individualized risk assessment [29]. The causal relationship between exercise and SCD in mild to moderate HCM remains unproven [8]. Currently, there is little evidence to indicate that moderate exercise represents a significant risk to children with mild to moderate HCM, and it does provide hemodynamic and psychological benefits [8,19,25,29]. Thus, shared decision-making with clinicians and families considering the benefits of mild- to moderate-intensity exercise, including improved cardiorespiratory fitness, physical functioning, and quality of life, is recommended [8,19,29]. While prior American Heart Association (AHA) recommendations advised against high-intensity sports for children with HCM symptoms or known sarcomere mutations, there is limited evidence to support significant risks from moderate exercise in patients without arrhythmia, LVOT obstruction, or exercise-induced complications [8,19]. The 2023 European Society of Cardiology (ESC) and AHA guidelines suggest that asymptomatic patients with early-stage HCM do not require clinical management or exercise restrictions [13,19].

## 7. Children Who Progress to Heart Failure

HCM in children may progress to end stage heart failure and require advanced heart failure therapies [2,6,8,9,24]. In a multicenter cohort study, the five-year survival rate for children diagnosed with HCM was found to be 71.1%, with heart failure being the leading cause of death [13]. Long-term studies have shown that children with HCM are twice as likely to require heart transplantation or left ventricular assist devices compared to adults [13]. In addition, there is accumulating evidence that heart failure in children differs in important ways from that in adults, and children lack many of the comorbid conditions present in adults [8]. End-stage heart disease in adults is commonly characterized by cardiac dilatation, wall thinning, and systolic dysfunction of the left ventricle, but this is not common in younger children [19,29]. Children tend to experience a more rapid decline than adults once end-stage heart failure develops and have a disproportionately elevated risk for early development of secondary pulmonary hypertension [4,16].

Managing heart failure in children presents significant challenges due to the lack of robust evidence, with current guidelines largely based on expert consensus and adult goal-directed therapies [2,11,19]. Relative to adult heart failure populations, inadequate statistical power associated with small sample sizes, phenotypic heterogeneity, limited observational periods, and age-specific variation in pharmacokinetics and pharmacodynamics have resulted in a lack of evidence for efficacy in children [19,40]. For example, ACE inhibitors and beta-blockers used for dilated cardiomyopathy in children have not been shown to improve transplantation-free survival, as demonstrated in a randomized trial involving 161 children [19]. Additionally, although medical therapies in the end stage of heart failure, such as diuretics and ACE inhibitors, may offer benefit, heart transplantation remains the ultimate treatment for children with advanced and end-stage heart failure [8,16,29]. Heart transplantation is considered when HCM progresses to end-stage heart failure, and cardiomyopathies are the leading cause of heart transplantation in childhood [19,31].

## 8. Current and Future Research Areas

### 8.1. Recommendations for Genotype-Positive Phenotype-Negative HCM in Children

Children with HCM exhibit a wide range of symptom severity, from asymptomatic to severe manifestations. There is no standardized treatment approach for these individuals. Efforts are underway to categorize patients based on their symptom expression and genotype to tailor treatment more effectively to the individual [8,19,29]. For children who have a positive genetic test but are not yet expressing a phenotype, there is considerable interest in identifying disease mitigation interventions for asymptomatic individuals genetically at risk for HCM [2,3,4,19]. A study of children with established HCM-related sarcomeric mutations but normal left ventricular wall thickness found that diltiazem may prevent the decrease in left ventricular cavity size and improve calcium handling in HCM [2]. In addition, the ARB valsartan was shown in a mouse model of HCM to inhibit transformative growth factor-beta (TGF-beta) activation, which has been implicated in the pathophysiology in left ventricular hypertrophy and fibrosis. The VANISH study showed that high-dose valsartan improved cardiac structure, function, and remodeling over 2 years in individuals with early-stage sarcomeric HCM, highlighting its potential for disease-modifying treatments in HCM [2,37,38]. Further support for treatment of genotype-positive phenotype negative children is evidenced by a case study of a Texas children’s hospital whose policy of treating all pediatric patients with beta-blockers at diagnosis showed a 15-year survival rate of 82%, as compared to only 60% 5-year survival in childhood HCM patients with no specific therapy [16]. Currently, there is insufficient evidence to support or refute initiating preventive or empirical treatment with beta-blockers, CCBs, or other medications [16]. Typically, this decision rests with experienced physicians and is determined on a case-by-case basis. Young, asymptomatic patients with HCM or those carrying mutations generally have a low risk of complications; therefore, in most cases, medical treatment is not initiated until symptoms are evident [2,3,5].

### 8.2. Management of Syndromic and Metabolic-Associated HCM

Although cause-specific therapies remain limited, the growing availability of disease-targeted treatments underscores the importance of precise diagnosis. Current management of syndromic and metabolic HCM follows clinical practice guidelines, with additional targeted therapies available for specific conditions [12]. Enzyme replacement therapy has demonstrated effectiveness in slowing or reversing disease progression in glycogen and lysosomal storage disorders by reducing cardiac mass, reversing remodeling, and improving cardiac function [2,12]. Gene therapy, including single-gene therapy and adeno-associated virus vectors, has shown promise in preclinical studies, with evidence of improved glycogen clearance and cardiac function in conditions like Pompe disease, Cori–Forbes disease, and Barth syndrome [6,10,12,19]. In patients with mitochondrial disorders, heart transplantation may be considered if cardiomyopathy is severe and extracardiac manifestations are mild or non-progressive [12]. Additionally, stem cell replacement therapy is being investigated as a potential treatment for lysosomal storage disorders [2]. Children with syndromic forms of HCM, such as those associated with RASopathies, are generally managed according to standard guidelines [12]. However, management is often more complex than in non-syndromic cases due to multisystem involvement, which can affect growth, metabolism, and other organ systems, and may contribute to a higher incidence of heart failure and mortality [40]. Many of these children also present with additional cardiac abnormalities, including pulmonary valve stenosis, mitral valve dysplasia, and atrial or ventricular septal defects, further complicating treatment. While standard strategies are applied, care must be individualized to account for life-threatening comorbidities and potential drug interactions [12,40]. Targeted therapies are beginning to show promise in this population. The MEK inhibitor trametinib has demonstrated beneficial effects in children with RASopathy-associated HCM, including improvements in cardiac hypertrophy, lower incidence of surgical intervention for LVOT obstruction and heart transplantation, and decreased mortality [40].

### 8.3. Risk Prediction Models of Childhood HCM

In response to the unpredictable nature of childhood HCM, there is growing effort to develop—specific risk stratification models for childhood-onset HCM to estimate SCD risk and guide decisions about ICD placement. These tools aim to improve clinical outcomes by balancing the potential life-saving benefit of ICDs against the risks and burdens of device implantation in children [4,7,8,10,24,39,41]. Unlike in adults, where risk markers and stratification tools are more established children present unique challenges due to age-related variability in symptoms, physiology, and disease progression [24,29,41]. A major limitation in the development of risk prediction models is that the accuracy of individual risk factors—particularly in children—remains debated [4,9,29,39,41]. Historically, childhood risk assessment relied heavily on extrapolated adult data due to a lack of large, dedicated child cohorts [19,41]. Only recently have child-specific risk models been developed, but these tools remain limited in their utility by small sample sizes, short follow-up periods, and ongoing uncertainty around age-specific risk markers [41].

**Figure 3:**
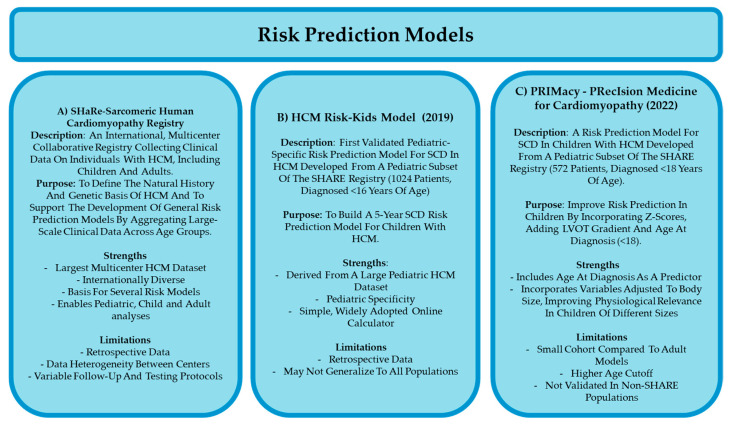
Summary of the SHaRe registry, HCM Risk-Kids, and PRIMaCY risk prediction models. (**A**) Outline of key aspects of the Sarcomeric Human Cardiomyopathy Registry (SHaRe), a large international dataset of genetically characterized HCM patients, including children. The registry provided the foundation for two pediatric-specific sudden cardiac death (SCD) risk prediction models: (**B**) HCM Risk-Kids, developed using SHaRe data from 1024 children with non-syndromic HCM to predict 5-year SCD risk, and (**C**) PRIMaCY, a subsequent model designed to address limitations of HCM Risk-Kids by incorporating a broader pediatric population and additional clinical variables.

The Sarcomeric Human Cardiomyopathy Registry (SHaRe) (Figure 3A) was established to create a large, international database capturing longitudinal clinical, genetic, and outcomes data from individuals with sarcomeric HCM across the lifespan. This registry contains historical events before SHaRe entry, demographics, genetics, clinical phenotype, and longitudinal outcome data [8,9]. Its primary purpose was to define the natural history and genetic basis of HCM and to support the development of general risk prediction models, as well as identify any unique aspects of certain populations with HCM [8,19]. However, SHaRe was not specifically designed for children, limiting its utility in childhood-onset disease.

In 2019, the first validated child-specific risk model was developed: HCM Risk-Kids (Figure 3B), based on data from 1024 children with non-syndromic childhood-onset HCM with the explicit goal of developing a validated, pediatric-specific risk prediction tool for SCD [7,8,19,20,32]. Using data from the SHaRe registry and incorporating age-specific clinical variables, HCM Risk-Kids fills a critical gap by providing the first model tailored specifically to children [7,8,19,32,41]. A study published in the European Heart Journal in 2021 externally validated the HCM Risk-Kids model in a large, geographically diverse cohort of 421 pediatric patients aged 1–16 years. The study found that a 5-year predicted risk threshold of ≥6% identified over 70% of SCD events, confirming the model’s utility in individualized risk prediction in children with HCM [7,8,41,42]. Building on this foundation, the PRIMaCY (PRecIsion Medicine for Cardiomyopathy) (Figure 3C) model was developed in 2022. It is derived from the pediatric subset of the SHaRe registry. PRIMaCY aimed to refine and personalize risk prediction by incorporating Z-score-normalized variables, LVOT gradient, and age at diagnosis, thereby capturing the heterogeneity of childhood physiology and development to improve individualized prognostication in children with HCM [20,32]. A study published in Europace in 2023 evaluated the clinical impact of using the PRIMaCY model in a cohort of 301 pediatric HCM patients. Here, they found that PRIMaCY had a similar discriminatory ability to identify adverse events in children, however PRIMaCY identified more children as meeting the threshold for ICD implantation, suggesting that some may receive an ICD unnecessarily [43]. These risk prediction models aim to improve the identification of patients at risk for adverse outcomes by weighing the predictive value of specific genotypes and clinical features; however, a major limitation remains the lack of consensus and robust data on the prognostic significance of many individual clinical presentations [7,8,20,32]. For example, specific gene mutations are often associated with extreme phenotypes [17]. However, inclusion of genotype in the PRIMaCY model did not significantly improve ability to predict the five-year risk of SCD in children diagnosed with HCM. These findings could be due, at least in part, to lack of consensus of which factors confer the highest risk of poor outcomes [5,8]. Additionally, these studies did not consider the role of unidentified genetic and epigenetic modifiers in HCM [5,8,15,24,25], or incomplete phenotypic expression of gene variant carriers [15,20]. However, current use of genetic data in risk prediction models has only increased predictive ability of models slightly at best [8,29] and ultimately, the role of genotypes in risk stratification for childhood HCM remains to be determined [5,8,23,29].

Taken together, current investigations are assessing the validity of risk prediction models in children, including HCM Risk-Kids and PRIMaCY, for application in clinical decision-making; however, the majority of these studies are retrospective, and the models are not yet sufficiently validated to guide management independently of clinical judgment.

Current guidelines from the ESC and AHA use a cumulative risk factor approach to recommend ICD implantation in children with HCM, however this approach is based on clinical risk factors largely extrapolated from adult practice and further validation revealed this approach showed only modest discriminatory, leading to unnecessary ICD implantation in many children [8,19].

Finally, there are very little data on risk stratification in patients with syndromic disease [8]. In a recent validation study, the HCM Risk-Kids model performed poorly in children with RASopathy-associated HCM, suggesting that existing tools may not be reliable in this group. Instead, clinical features such as unexplained syncope and non-sustained ventricular tachycardia appeared to be stronger predictors of adverse outcomes [44]. However, there is data emerging to suggest that SCD can occur in children and teenagers with RASopathy syndromes and inborn errors of metabolism. The limited available data suggests that the degree of left ventricular hypertrophy may be a strong predictor of SCD in patients with RASopathy syndromes, but future studies will need to address the lack of risk stratification models for syndromic patients and to identify disease-specific risk factors [8,19].

### 8.4. Future of HCM Research in Children

Future research in childhood-onset HCM explores multiple avenues to improve treatment outcomes in children. Investigations include preclinical studies targeting specific genetic subtypes to develop therapies tailored to underlying molecular mechanisms, clinical trials evaluating the efficacy and safety of interventions commonly used in adults adapted for children, and efforts to validate adult diagnostic tools for use in children to enhance early detection. While genotype-specific therapies remain largely in the preclinical stage, ongoing work in both preclinical and clinical settings is expanding the possibilities for more effective and precise management of childhood HCM.

#### 8.4.1. Preclinical Studies

Utilizing animal models of childhood-onset HCM may offer a unique approach to investigate novel treatments in humans and strengthen our understanding of pathological mechanisms in humans. A mouse model of sarcomeric HCM treated with diltiazem prior to phenotypic onset showed alleviation of hypertrophy, fibrosis, and myocyte disarray than placebo-treated mice [38]. A clinical trial in phenotype negative, genotype positive children and young adults is currently in progress [43]. Additional animal models of sarcomeric-youth-onset HCM have found success with inhibition of TGF-beta in a mouse model of HCM [5,23]. Atorvastatin has also been effective in animal models of youth-onset HCM [3]. Interestingly, CRISPR/Cas9 gene editing technology has shown efficacy in correcting an HCM-related genetic mutation before birth [6,32]. In this study, researchers targeted a pathogenic gene mutation in the MYBPC3 gene in sperm, and effectively reduced genetic transmission by 72% [6,32]. Despite these impressive results, direct genomic manipulation in early embryogenesis is unlikely to be widely adopted [6,10,32].

#### 8.4.2. Current Clinical Trials

Current HCM treatments focus on reduction in mechanical stress on the heart through symptom management as opposed to directly targeting pathophysiological pathways [5,16]. Myosin inhibitors have attracted interest as a potential mediator of mechanisms underlying HCM. Mavacamten and Aficamten have been tested in recent studies of HCM treatment [13,19,25,32]. Myosin inhibitors reduce contractility by acting on cardiac beta-myosin, thus directly targeting the hypercontractility phenotype observed in HCM [32]. Mavacamten has demonstrated efficacy in adults, including reduced LVOT pressure and improved clinical outcomes such as heart failure symptoms and functional status [13,17,25]. While these drugs are still under investigation, they offer hope for future treatment options in children with HCM [13,19]. Mavacamten has advanced to phase III clinical trials in humans and has shown benefits for LVOT obstruction, however it is not currently being studied in asymptomatic humans as means of HCM prevention [5,32]. Aficamten, which has a larger therapeutic window and shorter half-life than mavacamten, has also entered clinical trials with some promising results in decreasing LVOT obstruction and associated symptoms [25].

#### 8.4.3. Validation of Diagnostic/Prognostic Tools in Children

There is growing interest in validation of tools currently utilized in adult populations and their potential utility in children. Cardiac MRI withLGE is increasingly used to assess myocardial fibrosis, a strong predictor of SCD in adults with HCM, though its prognostic value in children remains unclear [2,5,8,30,32,45,46]. Gadolinium, an extracellular contrast agent, highlights scarred myocardial tissue by lingering in areas of fibrosis where extracellular space is expanded due to damaged or dead cardiomyocytes. This delayed washout effect allows LGE to identify fibrosis, which has been linked to increased arrhythmia risk and disease progression [8,22,24,32,46]. While studies suggest a correlation between LGE and left ventricular hypertrophy severity in children, further research is needed to validate its potential in risk stratification for children [8,30,32,45,46].

## 9. Conclusions

Childhood-onset HCM represents a significant but underrecognized healthcare burden. Though distinct from adult-onset disease, it remains understudied, with considerable variability in presentation that complicates clinical decision-making [11,26]. This underscores the importance of integrating genotype, phenotype, and individual risk factors when evaluating and managing child patients. Early detection and proactive management can allow many affected children to achieve near-normal life expectancy. However, the current lack of child-specific research has led to a reliance on adult-derived treatment strategies, which may not fully account for age-related differences in disease progression or treatment response. The treatment of children is further complicated by the wide developmental variability in growth, hormones, activity, comorbidities, and drug metabolism, all of which influence disease presentation and therapeutic response. The absence of a standardized, evidence-based care framework forces clinicians to rely heavily on judgment rather than clear guidelines. These limitations highlight an urgent need for expanded research and the development of tailored therapies to improve outcomes in children [2,4,7,19].

## Abbreviations

The following abbreviations are used in this manuscript:

ACE	Angiotensin Converting Enzyme
AHA	American Heart Association (AHA)
ARBs	Angiotensin Receptor Blockers
CCBs	Calcium Channel Blockers
ECG	Electrocardiogram
ECHO	Echocardiography
ESC	European Society of Cardiology
HCM	Hypertrophic Cardiomyopathy
ICDs	Implantable Cardioverter-Defibrillators
LGE	Late Gadolinium Enhancement
LVOT	Left Ventricular Outflow Tract
MRI	Magnetic Resonance Imaging
MYBPC3	Myosin-Binding Protein C3
MYH7	Beta-Myosin Heavy Chain
PRIMaCY	PRecIsion Medicine for Cardiomyopathy
RAAS	Renin–Angiotensin–Aldosterone System
SCD	Sudden Cardiac Death
SHaRe	Sarcomeric Human Cardiomyopathy Registry
TGF-beta	Transforming Growth Factor Beta
TNNI3	Troponin I
TNNT2	Troponin T
VANISH	Valsartan for Attenuating Disease Evolution in Early Sarcomeric Hypertrophic Cardiomyopathy
